# GLP-2 Attenuates LPS-Induced Inflammation in BV-2 Cells by Inhibiting ERK1/2, JNK1/2 and NF-κB Signaling Pathways

**DOI:** 10.3390/ijms17020190

**Published:** 2016-02-04

**Authors:** Nan Li, Bo-Wen Liu, Wen-Zhi Ren, Ju-Xiong Liu, Su-Nan Li, Shou-Peng Fu, Ya-Long Zeng, Shi-Yao Xu, Xuan Yan, Ying-Jie Gao, Dian-Feng Liu, Wei Wang

**Affiliations:** College of Animal Science and Veterinary Medicine, Jilin University, Changchun 130062, China; ln657748288@gmail.com (N.L.); Bowenliucn@outlook.com (B.-W.L.); rwz1964@163.com (W.-Z.R.); juxiong@jlu.edu.cn (J.-X.L.); lsnlhh2006@163.com (S.-N.L.); shoupengfu@163.com (S.-P.F.); zengyalong@foxmail.com (Y.-L.Z.); xushiyao1989@163.com (S.-Y.X.); yanxuan1992@outlook.com (X.Y.); yjgao@jlu.edu.cn (Y.-J.G.); ccdf@163.com (D.-F.L.)

**Keywords:** glucagon-like peptide-2, microglia, Parkinson’s disease, MAPK, NF-κB

## Abstract

The pathogenesis of Parkinson’s disease (PD) often involves the over-activation of microglia. Over-activated microglia could produce several inflammatory mediators, which trigger excessive inflammation and ultimately cause dopaminergic neuron damage. Anti-inflammatory effects of glucagon-like peptide-2 (GLP-2) in the periphery have been shown. Nonetheless, it has not been illustrated in the brain. Thus, in this study, we aimed to understand the role of GLP-2 in microglia activation and to elucidate the underlying mechanisms. BV-2 cells were pretreated with GLP-2 and then stimulated by lipopolysaccharide (LPS). Cells were assessed for the responses of pro-inflammatory enzymes (iNOS and COX-2) and pro-inflammatory cytokines (IL-1β, IL-6 and TNF-α); the related signaling pathways were evaluated by Western blotting. The rescue effect of GLP-2 on microglia-mediated neurotoxicity was also examined. The results showed that GLP-2 significantly reduced LPS-induced production of inducible nitric oxide synthase (iNOS), cyclooxygenase-s (COX-2), IL-1β, IL-6 and TNF-α. Blocking of Gα_s_ by NF449 resulted in a loss of this anti-inflammatory effect in BV-2 cells. Analyses in signaling pathways demonstrated that GLP-2 reduced LPS-induced phosphorylation of ERK1/2, JNK1/2 and p65, while no effect was observed on p38 phosphorylation. In addition, GLP-2 could suppress microglia-mediated neurotoxicity. All results imply that GLP-2 inhibits LPS-induced microglia activation by collectively regulating ERK1/2, JNK1/2 and p65.

## 1. Introduction

Parkinson’s disease (PD) is a common chronic neurodegenerative disease in humans, affecting millions of people all over the world [1]. The major character of PD’s neuropathology is the slow and progressive degeneration of dopaminergic neurons, which are located in the substantia nigra par compacta (SNpc) of the midbrain [2,3]. The loss of dopaminergic neurons results in severe and disabling clinical symptoms including akinesia, rigidity, tremor and some non-motor symptoms, which include cognitive impairments, sleep dysfunction and so on [4,5,6]. Although the exact mechanisms of PD pathogenesis are not defined, several factors have been implicated in the death of dopaminergic neurons, including mitochondrial dysfunction, excitotoxicity, and oxidative stress [7,8,9,10]. In recent years, a large number of studies have shown that the involvement of neuro-inflammation processes in the degeneration of dopaminergic neurons is important [1,11,12,13]. Microglia are the main effector cells in the process of neuro-inflammation. Uncontrolled over-activation of microglia is a major component of neuro-inflammation [14]. It has been suggested that several pro-inflammation cytokines and/or pro-inflammatory enzymes, which are produced by over-activated microglia, are believed to contribute to neurodegenerative processes [15,16,17]. Therefore, inhibition of microglial over-activation would be a promising strategy to alleviate the further progression of PD.

Glucagon-like peptide-2 (GLP-2) is a 33-amino-acid peptide hormone, which is derived from proglucagon [18]. Tissue-special processing of proglucagon can produce some biologically active peptides, such as GLP-1, GLP-2 and glucagon [19]. GLP-2 is a pleiotropic hormone synthesized in intestinal L-cells. Several studies have shown that GLP-2 improves mucosal blood flow and nutrient absorption, promotes the growth of the small intestine, decreases mucosal permeability, improves gut barrier function, and shows therapeutic efficacy in experimental models of both small and large bowel inflammation [18]. In the central nervous system, GLP-2 has been detected in the rat brainstem and hypothalamus [20]. In contrast to the increasing number of studies describing the enterotrophic role of GLP-2, much less is known about the potential functions of GLP-2 in the brain. Excitingly, previous studies have shown that GLP-2 has anti-inflammatory effects in the periphery [21,22,23]. Nonetheless, the anti-inflammatory effect of GLP-2 has not been illustrated in the brain. Herein, we hypothesize that GLP-2 has the potential to act directly on microglia to inhibit microglial over-activation, and then alleviate further progression of PD.

In the present study, we attempted to elucidate the anti-inflammatory potential of GLP-2 on the inflammatory response induced by lipopolysaccharide (LPS) in murine microglial BV-2 cells. To further investigate the underlying mechanisms, the involvement of NF-κB and MAPKs was also examined. The present study provides information revealing GLP-2 as a potential candidate compound with anti-inflammatory actions and suggests a scientific basis for further investigation of GLP-2 against neuro-inflammatory conditions.

## 2. Results

### 2.1. GLP-2 Inhibits LPS-Induced Expression of iNOS and COX-2 Proteins and mRNA in BV-2 Cells

**Figure 1:**
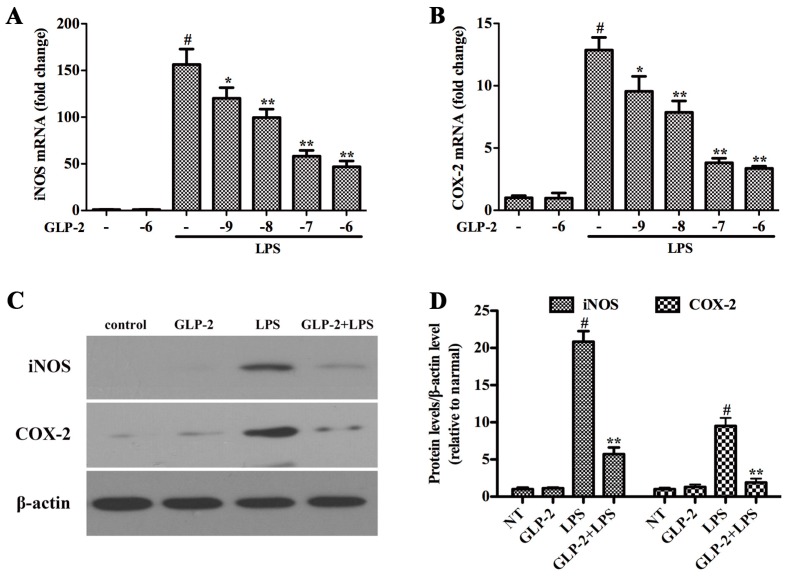
Glucagon-like peptide-2 (GLP-2) attenuates lipopolysaccharide (LPS)-induced iNOS and COX-2 in BV-2 cells. (**A**,**B**) The mRNA expression of iNOS and COX-2. BV-2 cells were pretreated with GLP-2 (10^−9^, 10^−8^, 10^−7^, and 10^−6^ M) 1 h prior to incubation of LPS (1 μg/mL) for 4 h; (**C**,**D**) Western blotting analysis of iNOS and COX-2. BV-2 cells were pretreated with 10^−6^ M GLP-2 for 1 h followed by LPS treatment at 1 μg/mL for 4 h. The mRNA and protein levels of iNOS and COX-2 normalized to β-actin. Each value was then expressed to non-treated (NT) control group, which was set as 1.00. Results are expressed as mean ± SD for each group from three independent experiments. ^#^ Significant compared with NT, *p* < 0.05; * *p* < 0.05 and ** *p* < 0.01 *versus* the GLP-2-untreated LPS-stimulated group.

iNOS and COX-2 are two important pro-inflammatory proteins correlated with LPS stimulation in microglia [13]. In order to investigate the effect of GLP-2 on the activation of LPS-stimulated BV-2 cells, BV-2 cells were pretreated with GLP-2 (10^−9^, 10^−8^, 10^−7^, 10^−6^ M) for 1 h and stimulated with LPS (1 μg/mL) for another 4 h. iNOS and COX-2 were examined by quantitative real-time PCR. GLP-2 notably dose-dependently inhibited the increased mRNA expression of iNOS (Figure 1A) and COX-2 (Figure 1B) stimulated by LPS. Pretreatment with GLP-2 (10^−6^ M) also significantly inhibited LPS-induced protein of iNOS (Figure 1C,D) and COX-2 (Figure 1C,D).

### 2.2. GLP-2 Attenuates Expression of Pro-Inflammatory Cytokines in LPS-Induced BV-2 Cells

**Figure 2:**
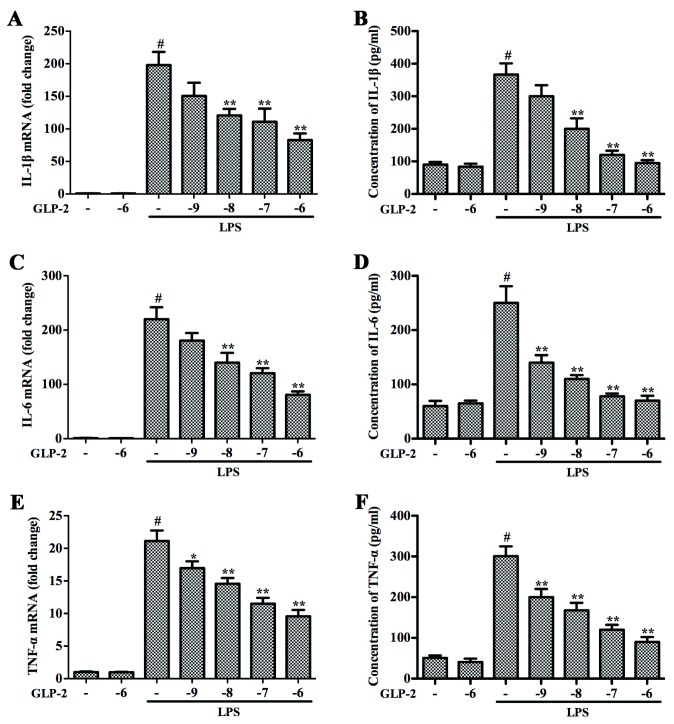
Effects of GLP-2 on LPS-induced expression of proteins and mRNA for IL-1β, IL-6 and TNF-α in BV-2 cells. BV-2 cells were pretreated with GLP-2 (10^−9^, 10^−8^, 10^−7^, and 10^−6^ M) 1 h prior to incubation of LPS (1 μg/mL) for 4 h (mRNA) or 24 h (protein). Proteins and mRNA of IL-1β (**A**,**B**); IL-6 (**C**,**D**) and TNF-α (**E**,**F**) were determined by quantitative real-time PCR and ELISA. Results are expressed as mean ± SD for each group from three independent experiments. ^#^ Significant compared with control (NT) alone, *p* < 0.05; * *p* < 0.05 and ** *p* < 0.01 *versus* the GLP-2-untreated LPS-stimulated group.

IL-1β, IL-6 and TNF-α are the main pro-inflammatory cytokines in the inflammatory process; they play important roles in the process of inflammation [24,25]. To investigate whether GLP-2 represses the production of these pro-inflammatory cytokines, BV-2 cells were pretreated with GLP-2 (10^−9^, 10^−8^, 10^−7^, 10^−6^ M) for 1 h and stimulated with LPS (1 μg/mL) for another 4 h. As shown in Figure 2, the significant increase of gene expression and protein secretion of IL-1β (Figure 2A,B), IL-6 (Figure 2C,D), and TNF-α (Figure 2E,F) resulting from the LPS stimulation was inhibited by GLP-2 in a dose-dependent manner in BV-2 cells. In order to determine the cytotoxicity of GLP-2, we investigated the dose effect of GLP-2 on cell viability by MTT (3-(4,5-dimethylthiazol-2-yl)-2,5-diphenyltetrazolium bromide) assay. BV-2 cells were incubated with various doses of GLP-2 for 24 h. The results of the MTT assay showed that GLP-2, even at a high concentration of 10^−4^ M, did not affect cell viability (data did not show), demonstrating that GLP-2 in non-cytotoxic levels in our experiments suppressed LPS-induced inflammatory responses in microglia via attenuating the expression of iNOS, COX-2 and pro-inflammatory cytokines.

### 2.3. GLP-2 Inhibits LPS-Induced Inflammation Responses through the Gα_s_ Signaling Pathway

**Figure 3:**
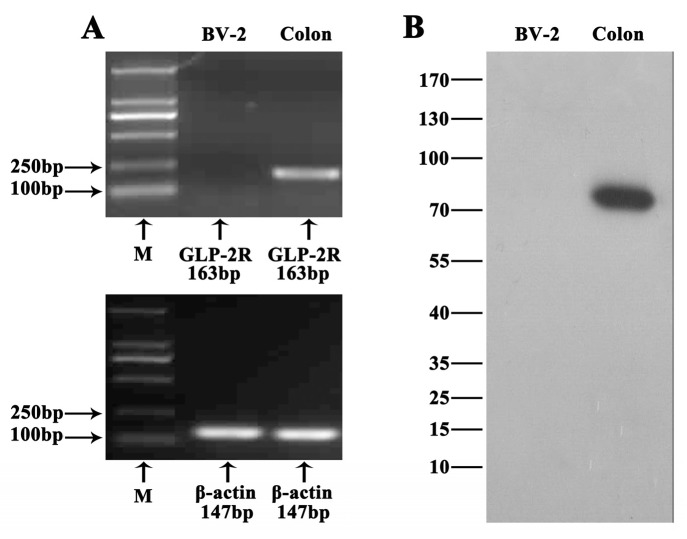
Expression of GLP-2R in BV-2 cells. (**A**) RT mixtures from colon (positive control) and BV-2 cells were analyzed to detect GLP-2R mRNA expression by PCR. The PCR products were visualized by 2% agarose gel electrophoresis. GLP-2R was detected in colon but not in BV-2 cells; (**B**) Western blotting of GLP-2R in colon and BV-2 cells. GPR109A was detected in colon but not in BV-2 cells.

**Figure 4:**
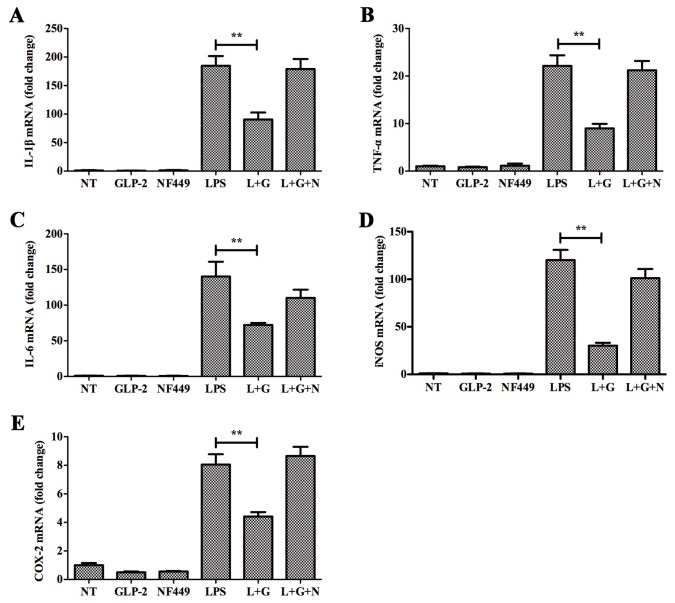
Effects of GLP-2 mediated by Gα_s_ signaling pathway. BV-2 cells were pretreated with vehicle or NF449 for 1 h. Medium was removed and replaced with vehicle, GLP-2 (10^−6^ M), NF449 (10 μM), LPS (1 μg/mL), LPS + GLP-2 (L + G), or LPS + GLP-2 + NF449 (L + G + N). BV-2 cells were sampled at 4 h. The mRNA of pro-inflammatory enzymes and pro-inflammatory cytokines were determined by quantitative real-time PCR. Attenuation by GLP-2 of LPS-induced mRNA of IL-1β (**A**); TNF-α (**B**); IL-6 (**C**); iNOS (**D**); and COX-2 (**E**) from BV-2 cells; this effect is abolished by pretreatment with NF449, ** *p* < 0.01.

**Figure 5:**
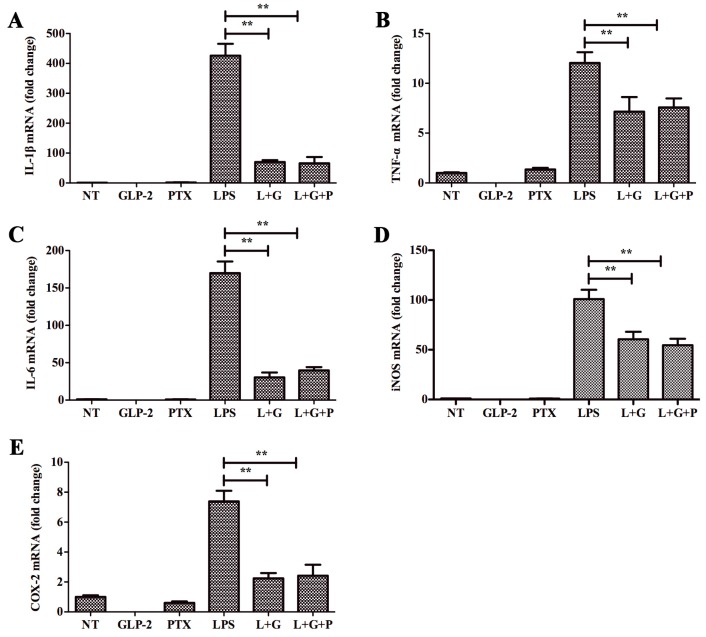
Effects of GLP-2 did not mediate by Gα_i/o_ signaling pathway. BV-2 cells were pretreated with vehicle or PTX for 1 h. Medium was removed and replaced with vehicle, GLP-2 (10^−6^ M), pertussis toxin (PTX) (100 ng/mL), LPS (1 μg/mL), LPS + GLP-2 (L + G), or LPS + GLP-2 + PTX (L + G + P). BV-2 cells were sampled at 4 h. The mRNA of pro-inflammatory enzymes and pro-inflammatory cytokines were determined by quantitative real-time PCR. Attenuation by GLP-2 of LPS-induced mRNA of IL-1β (**A**); TNF-α (**B**); IL-6 (**C**); iNOS (**D**); and COX-2 (**E**) from BV-2 cells; this effect is not abolished with pretreatment with PTX, ** *p* < 0.01.

GLP-2R is the functional receptor of GLP-2 [18]. In order to investigate whether this receptor mediates the anti-inflammatory function of GLP-2, we firstly detected the expression of GLP-2R in BV-2. As shown in Figure 3, GLP-2R mRNA (Figure 3A) and protein (Figure 3B) were not detected in BV-2. Therefore, GLP-2R cannot mediate the anti-inflammatory function of GLP-2. Also, previous studies demonstrated that the expression of GLP-2R was not detected in the gastrointestinal epithelium. However, Rocha *et al.* [26] found that GLP-2 stimulates proliferation in intestinal epithelial cells (Caco-2 cells) by decreasing the intracellular cyclic AMP (cAMP) level, and this function could be inhibited by pertussis toxin (PTX). This implies that multiple GLP-2 receptors are expressed in different cell types. In order to investigate whether there is another receptor in BV-2 cells, the cells were preincubated with NF449 for 30 min, a selective Gα_s_ protein-coupled receptor antagonist [27]. This resulted in complete blockade of the anti-inflammatory activity of GLP-2 (Figure 4), whereas pretreatment with PTX, an inhibitor of the Gα_i/o_ protein coupled receptor, had no effect (Figure 5).

### 2.4. GLP-2 Suppresses LPS-Induced Phosphorylation of ERK1/2 and JNK1/2 in BV-2 Cells

**Figure 6:**
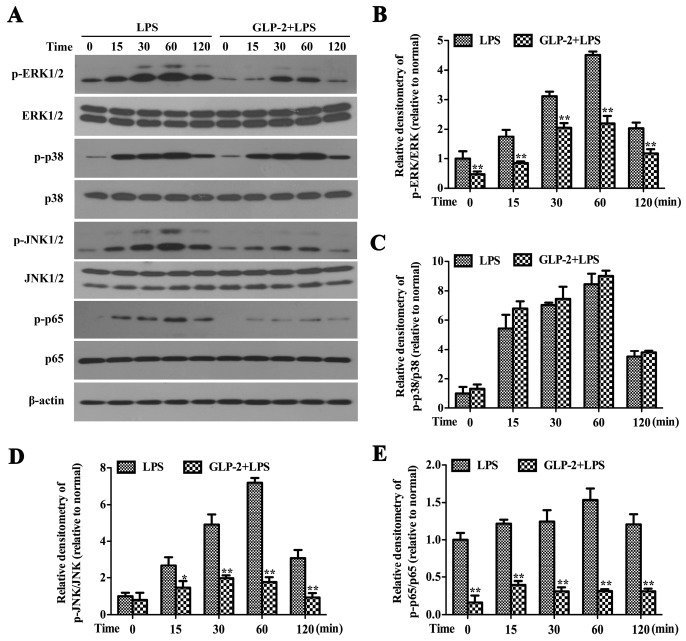
Effects of GLP-2 on LPS-induced activation of MAPKs and NF-κB in BV-2 cells. BV-2 cells were pretreated with GLP-2 (10^−6^ M) for 1 h followed by the treatment of LPS (1 μg/mL) for 0, 15, 30, 60 and 120 min. (**A**) Representative images of Western blotting for the phosphorylation of ERK1/2 (**B**); p38 (**C**); JNK1/2 (**D**) and NF-κB p65 (**E**). The activated levels of ERK1/2, p38, JNK1/2 and NF-κB p65 were quantified and normalized with their respective total protein. Each value was then expressed to control (LPS alone for 0 min), which was set as 1.00. * *p* < 0.05 and ** *p* < 0.01 *versus* treated with LPS alone within the same time point. Results are expressed as mean ± SD for each group from three independent experiments.

MAPK signaling pathways are known to play an important role in the regulation of inflammatory mediator production [28]. In order to investigate the relationship between the anti-inflammatory function of GLP-2 and these signaling pathways, we detected the effects of GLP-2 on the phosphorylation of ERK1/2, p38 and JNK1/2. BV-2 cells were stimulated with LPS (1 μg/mL) for 0, 15, 30, 60, 120 min in the presence or absence of GLP-2 (10^−6^ M). Cytoplasmic proteins were extracted, and the activation of ERK1/2, p38 and JNK1/2 was detected by Western blotting. The results showed that LPS (1 μg/mL) induced marked activation of ERK1/2, p38 and JNK1/2 in a time-dependent manner (Figure 6A). Pretreatment with GLP-2 in BV-2 cells markedly blocked LPS-induced activation of ERK1/2 (Figure 6A,B) and JNK1/2 (Figure 6A,D). However, the increased phosphorylation of p38 (Figure 6A,C) was not attenuated by GLP-2. The level of non-phosphorylated MAPK isoforms did not vary remarkably between groups.

### 2.5. GLP-2 Suppresses LPS-Induced Phosphorylation of p65 in BV-2 Cells

A number of studies have demonstrated that the NF-κB signaling pathway is a key mediator of inflammation, and its activation results in increased pro-inflammatory cytokines (TNF-α, IL-1β, and IL-6) and pro-inflammatory enzymes (iNOS and COX-2) [1,13]. To further investigate the mechanisms of GLP-2 on the inhibition production of pro-inflammatory mediator in BV-2 cells, we examined NF-κB signaling in response to LPS in BV-2 cells. BV-2 cells were stimulated with LPS (1 μg/mL) for 0, 15, 30, 60, 120 min in the presence or absence of GLP-2 (10^−6^ M). Cell lysates were subjected to Western blotting for phosphor-NF-κB p65, NF-κB p65, and β-actin. As shown in Figure 6, the level of active NF-κB p65 (phosphor-NF-κB p65) peaked at 60 min after LPS stimulation (Figure 6A,E). As expected, GLP-2 significantly reduced its level in BV-2 cells after LPS stimulation (Figure 6A,E).

### 2.6. GLP-2 Relieves Microglia-Mediated Neurotoxicity

**Figure 7:**
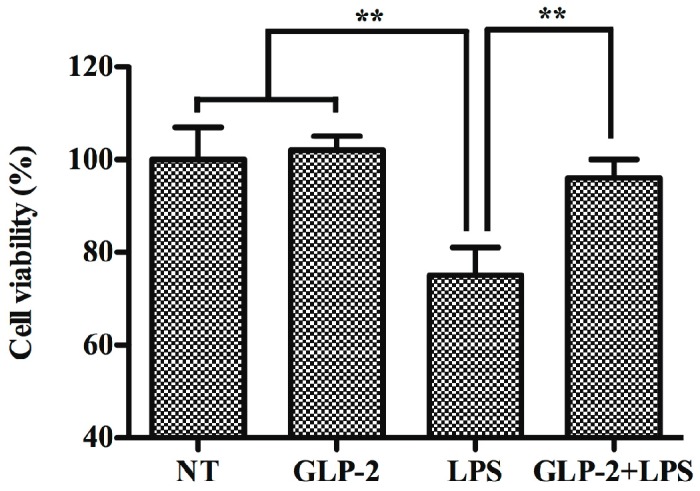
Effects of GLP-2 on microglia-mediated neurotoxicity. BV-2 cells were first treated with LPS (1 μg/mL) for 24 h in the presence or absence of GLP-2 (10^−6^ M). Then, the media were collected as conditioned media and added to PC12 cells. After 24 h, cell viability of PC12 cells was examined by MTT assay. Each value was then expressed as percentage of the control. The control group (NT) was set to 100%. ** *p* < 0.01. Results are expressed as mean ± SD for each group from three independent experiments.

Over-activation of microglia could induce dopaminergic neuron degeneration by producing pro-inflammatory mediators in PD [29,30]. So, we investigated whether GLP-2 relieves over-activated microglia-induced dopaminergic neuron degeneration. PC12 cells were used as a cell model to study PD. BV-2 cells were first treated with LPS (1 μg/mL) for 24 h in the presence or absence of GLP-2 (10^−6^ M). Then, the media were collected as conditioned media and added to PC12 cells. After 24 h, cell viability of PC12 cells was examined by MTT assay. The results showed that conditioned media from LPS-stimulated cells, but not from GLP-2 alone-treated cells, significantly increased cell death of PC12 cells (Figure 7). As expected, the conditioned media from BV-2 cells treated with LPS in the presence of GLP-2 (10^−6^ M) showed little neurotoxicity on PC12 cells (Figure 7). These results indicated that GLP-2 could suppress microglia-mediated neurotoxicity via inhibiting the expression of pro-inflammatory mediators.

## 3. Discussion

Several studies have demonstrated that neuro-inflammation is one of most important factors in the development of PD [31]. Microglia are the main effector cells in the process of neuro-inflammation. Over-activation of microglia is the main characteristic of neuro-inflammation. Over-activated microglia produce several neurotoxic factors such as pro-inflammatory cytokines and reactive oxygen species (ROS), which could cause dopaminergic neuron damage and even death [32]. Damaged or dead dopaminergic neurons can produce a number of substances, which can further activate microglia. Therefore, the inhibition of microglial over-activation serves as a key mechanism in the treatment of PD. The current study shows for the first time that GLP-2 had an anti-inflammatory function in murine microglia BV-2 cells. GLP-2 significantly inhibits LPS-induced enhancement of expression of pro-inflammatory enzymes (iNOS and COX-2) and pro-inflammatory cytokines (IL-1β, IL-6 and TNF-α) in BV-2 cells. The mechanistic study showed that the inhibitory effect of GLP-2 on BV-2 cells was mediated by the Gα_s_ protein-coupled receptor and involved the ERK1/2, JNK1/2 and NF-κB signaling pathways. In additional, we found that GLP-2 relieves the neurotoxicity result from the activated microglia.

GLP-2 is a 33-amino-acid peptide, which is highly conserved across different mammalian species [18]. Several studies proved that it is an intestinotrophic peptide, which is secreted by enteroendocrine L-cells in response to nutrient ingestion [19]. GLP-2 has several functions in the gastrointestinal tract, including increasing small and large intestinal weight via the stimulation of epithelial cell proliferation and the inhibition of apoptosis, enhancing absorptive surface area via enlarging crypts and villi, enhancing epithelial barrier capacity via decreasing transcellular and paracellular permeability, and so on. In recent years, although several studies prove that GLP-2 has anti-inflammatory properties, the underlying molecular mechanisms have not been resolved. In the central nervous system, GLP-2 has been detected in the rat brainstem and hypothalamus [20]. Over-activation of microglia is the key factor in neuro-inflammation, which is the one of the most important etiologies in PD. So, we detected the anti-inflammatory potential and mechanisms of GLP-2 on the inflammatory response induced by LPS in murine microglial BV-2 cells. Firstly, we found that GLP-2 significantly inhibited the protein and mRNA expression of pro-inflammatory enzymes (iNOS and COX-2) and pro-inflammatory cytokines (IL-1β, IL-6 and TNF-α) in BV-2 cells. These results suggest that GLP-2 has the potential to act directly on microglia to inhibit pro-inflammatory mediator production.

GLP-2R is a G protein-coupled receptor, which is the functional receptor of GLP-2 [18]. GLP-2R can initiate anti-apoptotic responses through the cAMP pathway involving both PKA-dependent and independent mechanisms [33]. The Central Nervous System (CNS) GLP-2R plays a key role in the control of glucose homeostasis and insulin sensitivity via the GLP2R-PI3K-mediated central melanocortin system [34]. Furthermore, GLP-2R can also couple to alternate G proteins Gα_s_ and Gα_i/o_ [35]. Gα_s_ activates the cAMP-dependent pathway by stimulating the production of cAMP from ATP; however, Gα_i_ inhibits the production of cAMP from ATP [36]. To investigate whether GLP-2R mediates the anti-inflammatory function of GLP-2, we firstly detected the expression of GLP-2R in BV-2 cells. Unfortunately, we did not detect GLP-2R mRNA and protein expression in BV-2 cells, suggesting that GLP-2 anti-inflammatory action in BV-2 cells is mediated by a distinct and yet unidentified receptor. Evidence that GLP-2 stimulates proliferation in Caco-2 cells which did not express GLP-2R, and that this function could be inhibited by PTX [26], points toward the existence of an unknown receptor distinct from the classical GLP-2R. Indeed, the results of our study suggested that the anti-inflammation function of GLP-2 - in BV-2 cells is mediated by a Gα_s_ protein receptor, because specific Gα_s_ but not Gα_i/o_ inhibitors prevented the anti-inflammatory effect of GLP-2.

NF-κB and the MAPK family including ERK1/2, p38 and JNK1/2 are important regulators of pro-inflammatory gene expression [37,38,39]. They could be phosphorylated by LPS in BV-2 cells, and then enhance the expression of pro-inflammatory enzymes (iNOS and COX-2) and pro-inflammatory cytokines (IL-1β, IL-6 and TNF-α) [13]. To investigate the molecular mechanisms underlying the anti-inflammatory effect of GLP-2, we examined whether GLP-2-mediated signaling pathways modulate NF-κB and MAPKs. Pretreatment with GLP-2 did not inhibit LPS-induced phosphorylation of p38, demonstrating that the anti-inflammatory property of GLP-2 is not mediated by p38 signaling. On the other hand, LPS-induced phosphorylation of ERK1/2, JNK1/2 and p65 were significantly inhibited by GLP-2 pretreatment, suggesting the anti-neuro-inflammation of GLP-2 is potentially produced via inhibiting the activation of ERK1/2, JNK1/2 and p65.

Over-activation of microglia could induce dopaminergic neuron degeneration by producing pro-inflammatory mediators in PD [29,30]. Our study proved that GLP-2 could inhibit the over-activation of microglia, and thus GLP-2 can play a role in the protection of dopaminergic neurons through its anti-inflammatory effect. Therefore, we investigated whether GLP-2 relieves neurotoxicity induced by activated microglia. Our results showed that GLP-2 could suppress microglia-mediated neurotoxicity via inhibiting the expression of pro-inflammatory mediators.

In conclusion, the results of this study provide evidence that GLP-2 exhibits its anti-inflammatory effects via a Gα_s_ protein receptor and suppressing activation of ERK1/2, JNK1/2 and p65. Furthermore, GLP-2 can relieve microglia-mediated neurotoxicity. This finding provides a new molecular insight into the mechanism by which GLP-2 exerts its anti-inflammatory function. Arising from the above, we suggest that GLP-2 may possibly be helpful in alleviating the progression of PD.

## 4. Materials and Methods

### 4.1. Cells and Treatments

The BV-2 cells (immortalized mouse microglial cell line) (Cell Bank of Chinese Academy of Sciences, Shanghai, China) and PC12 cells were seeded on 6 cm cell culture plates and maintained in DMEM (Hyclone, Logan, UT, USA) supplemented with 10% (*v*/*v*) FBS (Hyclone, Logan, UT, USA), 50 U/mL penicillin, and 50 μg/mL streptomycin at 37 °C in 5% CO_2_/95% atmosphere. The culture medium was changed twice a week and cultures were passaged at 80% confluence after trypsinization (0.05%, *w*/*v*). Changes in cell morphology and growth conditions were carefully monitored using an inverted microscope. BV-2 cells were precultured in serum-free DMEM for 4 h to reduce mitogenic effects. Cells were pretreated with various concentrations of GLP-2 (from Tocris Bioscience, Bristol, UK; or Sigma-Aldrych, Dorset, UK) for 1 h and stimulated with LPS (obtained from *Escherichia coli*, serotype O26:B6; Sigma-Aldrich, St. Louis, MO, USA).

### 4.2. Cell Viability Assay

Cell viability was determined by MTT assay. Briefly, BV-2 cells were initially seeded into 96-well plates at a density of 1 × 10^4^ cells/well. Following treatment, MTT (5 mg/mL) was added to each well and incubated at 37 °C for 4 h. After added 100 μL of DMSO to dissolve the crystals, the optical density was measured at 570 nm. Three replicates were carried out for the each different treatment.

### 4.3. RNA Extraction, Reverse Transcription and Quantitative Real-Time PCR

Total RNA was extracted from the cells using TRI Reagent (Sigma-Aldrich, St Louis, MO, USA), according to the supplier’s protocol. Total RNA was then treated with RNAse-free DNAse type I, quantified by measuring the absorbance at 260 and 280 nm and stored at −80 °C until analysis. The extracted RNA was subjected to RT-PCR using a PrimeScript RT Reagent Kit With gDNA Eraser (Takara Shuzo Co., Ltd., Kyoto, Japan). The mRNA levels of various genes were evaluated by quantitative polymerase chain reaction (qRT-PCR) using a SYBR Green QuantiTect RT-PCR Kit (Roche, South San Francisco, CA, USA), and each sample was assessed in triplicate. The relative expression levels of iNOS, COX-2, TNF-α, IL-1β, and IL-6 were calculated relative to β-actin (the normalizer) using the comparative cycle threshold method. The primer sequences for the tested genes are shown in Table 1.

**Table 1:** The primer sequences of β-actin, GLP-2R, iNOS, COX-2, TNF-α, IL-1β, and IL-6.

Gene	Sequences	Length (bp)
*β-actin*	(F) 5′-GTCAGGTCATCACTATCGGCAAT-3′	147
	(R) 5′-AGAGGTCTTTACGGATGTCAACGT-3′	
*GLP-2R*	(F) 5′-GGTCCTCCTGCACTTT-3′	163
	(R) 5′-CCAGGGAATAACAAACAGC-3′	
*iNOS*	(F) 5′-CACCCAGAAGAGTTACAGC-3′	186
	(R) 5′-GGAGGGAAGGGAGAATAG-3′	
*COX-2*	(F) 5′-AGAGTCAGTTAGTGGGTAGT-3′	170
	(R) 5′-CTTGTAGTAGGCTTAAACATAG-3′	
*TNF-α*	(F) 5′-CCACGCTCTTCTGTCTACTG-3′	145
	(R) 5′-GCTACGGGCTTGTCACTC-3′	
*IL-1β*	(F) 5′-TGTGATGTTCCCATTAGAC-3′	131
	(R) 5′-AATACCACTTGTTGGCTTA-3′	
*IL-6*	(F) 5′-AGCCACTGCCTTCCCTAC-3′	156
	(R) 5′-TTGCCATTGCACAACTCTT-3′	

### 4.4. ELISA

BV-2 cells were seeded in 24-well plates pretreated with various concentrations of GLP-2 for 1 h followed by stimulation with LPS (1 μg/mL) for another 24 h. After stimulation, culture media were collected and centrifuged at 13,000 rpm for 3 min. The amounts of TNF-α, IL-1β, and IL-6 in the culture medium were measured with commercial ELISA kits obtained from BioLegend (San Diego, CA, USA).

### 4.5. Western Blot Analysis

BV-2 cells were grown to 80% confluence, serum-starved for 4 h (reduce mitogenic effects), and then treated with GLP-2 (10^−8^ M) or vehicle. The cells were washed with ice-cold PBS and harvested at 5, 15, 30, 60, and 120 min using lysis buffer (Beyotime Inst. Biotech, Beijing, China) according to the manufacturer’s instructions. Concentration of the protein was measured using a bicinchoninic acid protein assay kit (Beyotime Inst. Biotech, Beijing, China). A total of 50 μg of protein was resolved by 12% SDS-polyacrylamide gel electrophoresis (SDS-PAGE) and transferred onto immunoblot polyvinylidene difluoride membranes (Chemicon International, Millipore, Billerica, MA, USA). The blots were blocked with 5% non-fat milk in Tris-buffered saline with 0.1% Tween (TBS-T) for 1 h, washed four times with TBS-T, and incubated overnight at 4 °C with primary antibodies against iNOS (1:2000), COX-2 (1:1000), (Abcam, Cambridge, CA, USA), phosphor-ERK1/2 (1:2000), ERK1/2 (1:2000), phosphor-p38 (1:2000), p38 (1:1000), phosphor-JNK1/2 (1:1000), JNK1/2 (1:2000), phosphor-NF-κB p65 (1:1000), NF-κB p65 (1:1000) (Cell Signaling Technology, Danvers, MA, USA), GLP-2R (1:500), and β-actin (1:2000) (Santa Cruz Biotechnology Inc., Santa Cruz, CA, USA). The blots were then washed four times for 15 min each in TBS-T and incubated with a horseradish peroxidase-labeled secondary goat anti-rabbit (1:2000) or rabbit anti-goat antibody (1:2000) (Santa Cruz Biotechnology Inc., Santa Cruz, CA, USA) for 1 h at room temperature. Next, the blots were washed again four times for 15 min each in TBS-T. Membranes were visualized with enhanced chemiluminescence (ECL kit; Applygen Inst. Biotech, Beijing, China).

### 4.6. Statistical Analyses

The data are presented as the mean ± SD and were analyzed using SPSS 12.0 statistical software package (SPSS Inc., Chicago, IL, USA). The groups were compared by one-way analysis of variance (ANOVA) followed by the least significant difference test. A *p* value of less than 0.05 was considered statistically significant.

## Abbreviations

PD: Parkinson’s disease; GLP-2: glucagon-like peptide-2 (GLP-2); LPS: lipopolysaccharide (LPS); PTX: pertussis toxin (PTX); SDS-PAGE: SDS-polyacrylamide gel electrophoresis.
